# CFHBA-PID Algorithm: Dual-Loop PID Balancing Robot Attitude Control Algorithm Based on Complementary Factor and Honey Badger Algorithm

**DOI:** 10.3390/s22124492

**Published:** 2022-06-14

**Authors:** Jianan Lin, Rongjia Zheng, Yirong Zhang, Jinkai Feng, Wei Li, Kaiqing Luo

**Affiliations:** 1 Guangdong Provincial Key Laboratory of Quantum Engineering and Quantum Materials, School of Physics and Telecommunication Engineering, South China Normal University, Guangzhou 510006, China; 20192332124@m.scnu.edu.cn (J.L.); 20192332113@m.scnu.edu.cn (R.Z.); 20192332008@m.scnu.edu.cn (Y.Z.); 20192332046@m.scnu.edu.cn (J.F.); liwei@m.scnu.edu.cn (W.L.); 2 Guangdong Provincial Engineering Research Center for Optoelectronic Instrument, School of Electronics and Information Engineering, South China Normal University, Foshan 528225, China

**Keywords:** metaheuristic algorithms, honey badger algorithm, complementary factor, CF-ITAE, Dual-loop PID control, balancing robot

## Abstract

The PID control algorithm for balancing robot attitude control suffers from the problem of difficult parameter tuning. Previous studies have proposed using metaheuristic algorithms to tune the PID parameters. However, traditional metaheuristic algorithms are subject to the criticism of premature convergence and the possibility of falling into local optimum solutions. Therefore, the present paper proposes a CFHBA-PID algorithm for balancing robot Dual-loop PID attitude control based on Honey Badger Algorithm (HBA) and CF-ITAE. On the one hand, HBA maintains a sufficiently large population diversity throughout the search process and employs a dynamic search strategy for balanced exploration and exploitation, effectively avoiding the problems of classical intelligent optimization algorithms and serving as a global search. On the other hand, a novel complementary factor (CF) is proposed to complement integrated time absolute error (ITAE) with the overshoot amount, resulting in a new rectification indicator CF-ITAE, which balances the overshoot amount and the response time during parameter tuning. Using balancing robot as the experimental object, HBA-PID is compared with AOA-PID, WOA-PID, and PSO-PID, and the results demonstrate that HBA-PID outperforms the other three algorithms in terms of overshoot amount, stabilization time, ITAE, and convergence speed, proving that the algorithm combining HBA with PID is better than the existing mainstream algorithms. The comparative experiments using *CF* prove that CFHBA-PID is able to effectively control the overshoot amount in attitude control. In conclusion, the CFHBA-PID algorithm has great control and significant results when applied to the balancing robot.

## 1. Introduction

PID control algorithms are the most prevailing and widely used control algorithms in the industry, agriculture, and new technology industries such as with drones and unmanned vehicles. In recent years, balancing robots have been developed rapidly [1,2], and wheeled balancing robots are more widely used in production because they are simpler and more stable to control than legged balancing robots, as well as being cheaper and easier to manufacture [3]. For a balancing robot, the most important thing is the stability and robustness of its attitude control, which depends on the control algorithm. The Dual-loop PID control algorithm [4] is often used in the control of balancing robots [5], while it is difficult to tune the PID parameters simply by manual. Many studies [6,7,8,9,10,11,12,13,14,15,16,17,18] have proposed different methods to optimize the PID algorithm, which enable the PID algorithm to have better performance and to be applied to various scenarios. The merit of the PID parameters determines the effectiveness of the control algorithm as well as the accuracy and stability of balancing robot control. Therefore, the present study aims at presenting a Dual-loop PID control algorithm for balancing robots capable of automatically tuning parameters.

With the continuous development of the methods for tuning PID parameter, they include manual PID parameter tuning methods and intelligent PID parameter tuning methods. Among them, using metaheuristic algorithms for PID parameter tuning has important research significance and development prospects. Metaheuristic algorithms [19] are optimization algorithms that use multiple agents and multiple iterations to obtain the optimal solution to a specific problem. Exploration and exploitation are indispensable components of metaheuristics that contradict each other, but both determine the merits of metaheuristics. In chronological order, metaheuristics can be divided into traditional metaheuristics and novel metaheuristics.

The classical traditional metaheuristics are the particle swarm optimization algorithm (PSO) [20], ant colony optimization algorithm (ACO) [21], and genetic algorithms (GAs) [22], which have been frequently used in the PID control algorithm in recent years. In [8], the PSO-PID control algorithm is proposed to achieve freeway ramp control regulation. The results show that the convergence speed of the PSO-PID control algorithm is faster than that of the BP neural network in the process of ramp control, thus obtaining the best ramp control. However, the PSO-PID control algorithm is not accurate in the search process and easily falls into the local optimum. In [9], the PSO algorithm is used for the parameter optimization of a chaotic synchronous PID controller, and the algorithm is applied to chaotic synchronous control with better results than the evolutionary programming (EP) algorithm. In [10], the PSO algorithm is used to tune the quantization and proportionality factors in the fuzzy PID, resulting in a self-tuning particle swarm fuzzy PID control algorithm for the control of the robotic arm. The optimized controller has higher control precision and better control input torque on the control of the robotic arm. In [11], a weighted cooperative PID and LQR control algorithm using the PSO algorithm and the FCPC control algorithm are presented to control the robotic arm. The FCPC control algorithm converges faster in the position control of the robotic arm compared to the single PID control algorithm and the LQR control algorithm. In [12], the NSGA-II-PID control algorithm is obtained by using the evolutionary algorithm NSGA-II to tune the PID parameters of the PID controller of the greenhouse climate control system, which achieved good control performance. The schemes of optimizing the PID control algorithm through the traditional metaheuristic algorithm solve the problem that the parameters of the traditional PID control algorithm are difficult to tune. However, due to the limitations of traditional metaheuristic algorithms, it may lead to premature convergence and local optimal solutions obtained during parameter tuning, which may produce invalid loops [23].

In recent years, more and more metaheuristics have been proposed that outperform traditional metaheuristics in terms of their ability to search for globally optimal solutions, like the Archimedes Optimization Algorithm (AOA) [24], Whale Optimization Algorithm (WOA) [25], Harris−Hawkes Optimization (HHO) [26], Atom Search Algorithm (ASO) [27]. In [13], a combination of atomic search algorithm (ASO) and logistic chaotic sequences is applied for fractional order PID (FOPID) control. The FOPID algorithm achieves a better step response of the control output and has a better suppression effect on disturbances. In [14], the Crow search algorithm is proposed for the combination of DC motor PID control algorithm, and the obtained transient response is superior to the PSO-PID control algorithm. In [15], an artificial bee colony (ABC) algorithm is used to optimize the PID/PI control algorithm in the automatic generation control of interconnected reheat thermal power systems, which has a stronger global search capability than the PSO-PID/PI control algorithm. In [16], a hybrid algorithm (D2AOFAT) combining the dynamic differential annealing optimization (DDAO) and feedback artificial tree (FAT) algorithm is proposed to tune the parameters of a high-order fractional-order PID control algorithm. The time-domain metrics of the responses obtained by this algorithm are better than the conventional algorithm. In [17], the Whale Optimization Algorithm (WOA) is applied to the PID controller which achieved the effective trajectory tracking control of the robot manipulator. In [18], the chaotic whale algorithm (CWOA) is proposed to optimize the parameter tuning of the FOPID control algorithm. The CWOA-FOPID control algorithm obtains better ITAE and ISE values during the optimization search process, and the time-domain metrics of the output response are better than those obtained by other existing controllers in the reverse osmosis desalination process. Optimizing the PID control algorithm through metaheuristic algorithms with better search capability avoids the problem of premature convergence to some extent. However, given that the global optimal solution is extremely complicated, the search capability and search speed of metaheuristic algorithms still need to be improved, and there is still much room for improvement in the performance of these control algorithms.

Due to the increasing complexity of real-life problems and the highly multimodal nature of the problems, many existing metaheuristic algorithms present local optimal solutions rather than global optimal solutions during the search process. Therefore, developing a metaheuristic algorithm that well balances exploitation and exploration has become the focus of metaheuristic algorithm research. In [28], the Honey Badger Algorithm (HBA) is proposed, which is inspired by the hunting process of honey badgers. HBA maintains a large enough population diversity during the search process and possesses a dynamic search strategy to simultaneously balance the exploration. These two properties of HBA play a very effective role in avoiding the effect of local optimum, which is conducive to better avoiding the problems of early convergence and local optimal solutions in the process of PID parameter tuning.

In the process of balancing robot attitude control, the merit of the Dual-loop PID parameters determines the accuracy and stability of balancing robot attitude control. From the comparative experimental results, it can be seen that for the complex PID control algorithm, the controller obtained by the metaheuristic algorithm with ITAE as fitness was not effective and has the problem of short adjustment time but large overshoot. Therefore, it is necessary to explore an optimization method for the Dual-loop PID parameter tuning that can balance the overshoot and response speed.

In this paper, an HBA Dual-loop PID control algorithm (CFHBA-PID algorithm) based on CF-ITAE for balancing robot attitude control is proposed. To verify the superiority of the HBA-PID algorithm, the AOA-PID, WOA-PID, and PSO-PID algorithms are applied to the attitude control of the balancing robot for comparison experiments. In addition, a new indicator CF-ITAE is proposed to be compared with ITAE. The CFHBA-PID algorithm, CFAOA-PID algorithm, CFWOA-PID algorithm, and CFPSO-PID algorithm are compared with the original control algorithm, so as to verify the superiority of CF-ITAE.

The main contributions of this paper are the following:

1. The HBA (Honey Badger Algorithm) is utilized in the Dual-loop PID algorithm parameter tuning for the first time, and the HBA-PID control algorithm is proposed to achieve stable and robust balancing robot attitude control.
2. A complementary factor (CF) is proposed and implemented as a hyperparameter in the new proposed CFHBA-PID algorithm, which can be adjusted according to the actual requirements to achieve the balance of overshoot and response speed during the process of searching the optimal parameters.
3. The CFHBA-PID algorithm is applied to the Dual-loop PID attitude control of the balancing robot, and achieves the best control compared to the existing mainstream algorithms.

## 2. Materials and Methods

### 2.1. PID

#### 2.1.1. PID Control Algorithm

**Figure 1:**
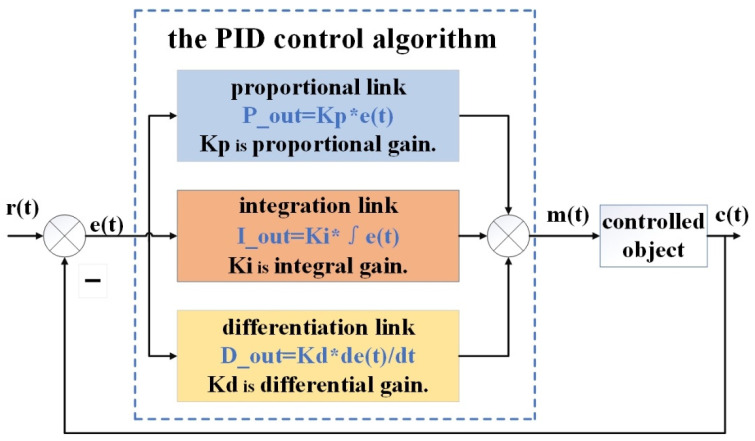
The basic model of the PID control algorithm.

Figure 1 presents the basic model of the PID control algorithm. The PID control algorithm is a series compensation control algorithm consisting of the proportional session, integral session, and differential session. In the PID controller, the error quantity  e(t)  is the difference between the system feedback quantity  c(t)  and the input quantity  r(t). The output control quantity m(t) is obtained by scaling up or down as well as integrating and differentiating the error quantity e(t). The output control quantity m(t) calculation equation is given as Equation (1):

(1) $$m\left(t\right)=K_{p}\times e\left(t\right)+K_{i}\times \int_{0}^{t}e\left(t\right)dt+K_{d}\frac{de\left(t\right)}{dt}$$

where t is the current time of the PID controller, $K_{p}$ is the proportional factor, $K_{i}$ is the integral factor, and $K_{d}$ is the differential factor. According to the PID control algorithm, $K_{p}$ plays a role in making the control timely, whereas too much $K_{p}$ will cause a loss of stability and too little $K_{p}$ will result in a loss of regulation, as well as inappropriate $K_{p}$ will cause static errors. $K_{i}$ plays a role in eliminating static errors, but it results in system oscillations, and the larger the $K_{i}$, the larger the integral effect. $K_{d}$ plays a role in overcoming the hysteresis of a controlled object, and the larger the $K_{d}$, the more potent the differential effect. Therefore, $K_{p}$, $K_{i}$, and $K_{d}$ play a decisive role in the performance of the control algorithm, and appropriate parameters can ensure a stable system with small errors.

#### 2.1.2. Dual-Loop PID Control Algorithm

**Figure 2:**
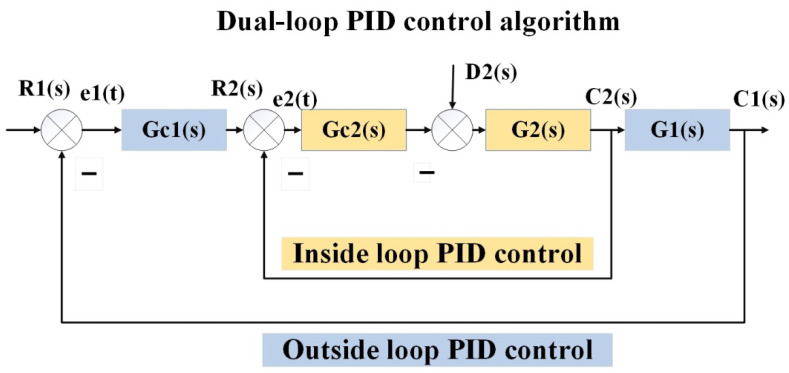
The basic model of the Dual-loop PID control algorithm.

Figure 2 illustrates the basic model of the Dual-loop PID control algorithm. The Dual-loop PID control algorithm is a dual closed-loop control system by combining two PID controllers, one of which acts as the outside loop of the system and the other acts as the inside loop of the system. The output of the outside loop PID controller affects the desired value of the inside loop PID controller, while the output of the inside loop PID controller affects the actual value of the outside loop, thus achieving Dual-loop control of the inside and outside loops. Compared with single-loop PID control, the Dual-loop PID algorithm increases the frequency of the control system operation and reduces the oscillation period and regulation time, resulting in enhanced system rapidity.

#### 2.1.3. Dual-Loop PID Algorithm for Attitude Control of Balancing Robots

**Figure 3:**
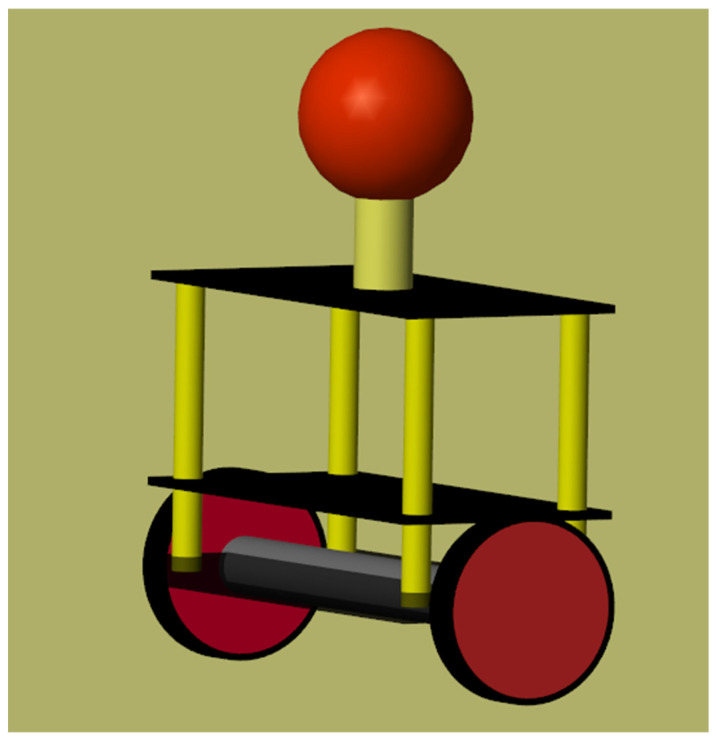
The model of the balancing robot.

**Figure 4:**
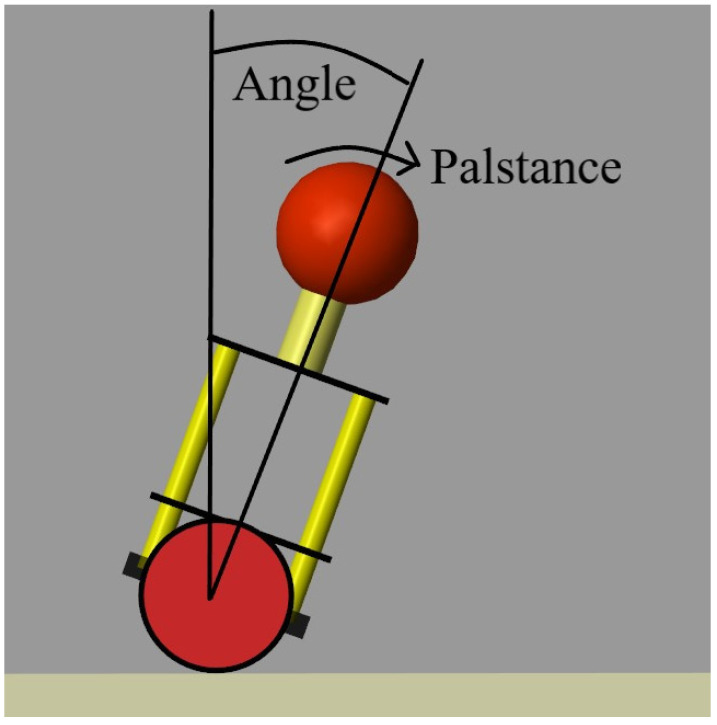
The cross-section of the balancing robot.

A balancing robot’s control relies on attitude control. The mechanical model of the balancing robot is shown in Figure 3, while the cross-section is shown in Figure 4. The outside loop of attitude control is the angular loop, which is obtained by using the angular error $e_{outside}\left(t\right)$ as the input quantity and the output quantity $C_{outside}\left(t\right)$ as the desired value of the inside loop. The equations of the outside loop control are given as Equations (2) and (3):

(2) $$e_{outside}\left(t\right)=Angle\_expect-Angle$$

(3) $$C_{outside}\left(t\right)=K_{p\_outside}\times e_{outside}\left(t\right)+K_{i\_outside}\times \int e_{outside}\left(t\right)dt\;+K_{d\_outside}\frac{de_{outside}\left(t\right)}{dt}$$

where Angle_expect is the desired angle, and Angle is the actual angle. $K_{p\_outside}$, $K_{i\_outside}$, and $K_{d\_outside}$ are parameters of the outside-loop PID controller.

The inside loop of the attitude control is the angle loop, which takes the angular velocity error $e_{inside}\left(t\right)$ as the input quantity and the output quantity $C_{outside}\left(t\right)$ as the force to balance the robot. The equations of the outside loop control are given as Equations (4) and (5):

(4) $$e_{inside}\left(t\right)=C_{outside}\left(t\right)-Palstance$$

(5) $$C_{inside}\left(t\right)=K_{p\_inside}\times e_{inside}\left(t\right)+K_{i\_inside}\times \int e_{inside}\left(t\right)dt\;+K_{d\_inside}\frac{de_{inside}\left(t\right)}{dt}$$

where Palstance is the actual angular velocity. $K_{p\_inside}$, $K_{i\_inside}$, $K_{d\_inside}$ are parameters of the inside-loop PID controller.

**Figure 5:**
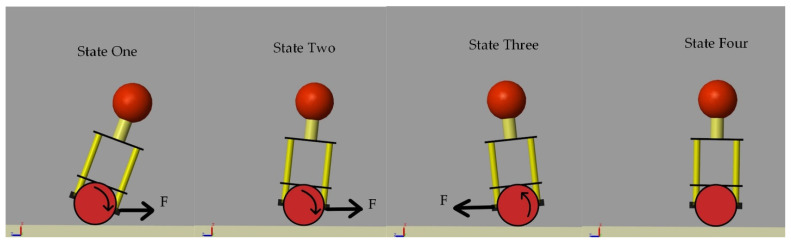
The state diagram of the balancing robot in attitude control.

Figure 5 shows the state diagram of the balancing robot in attitude control. State One is the initial state of the balancing robot. State Four is the upright state of the balancing robot. The balancing robot starts from State One and aims to achieve State Four. In order to reach State Four, the outer loop output $C_{outside}\left(t\right)$ is obtained through the outer loop Equations (2) and (3). Then, $C_{outside}\left(t\right)$ is used as the desired angular velocity value in the inner-loop Equations (4) and (5) to calculate the output of the inner-loop $C_{inside}\left(t\right)$. Finally, $C_{inside}\left(t\right)$ is used as the input of the balancing robot motor to generate the corresponding control force F to change the attitude of the balancing robot.

#### 2.1.4. PID Control Performance Indicators

In the present paper, the dynamic performance of the control system is evaluated from two aspects. In terms of time-domain metrics, the rise time $t_{r}$, transition process time $t_{s}$, and overshoot $\sigma_{p}$ are used to determine the superiority of the step response of the output. The rise time $t_{r}$ is the time it takes for the step response curve to rise from zero to the steady-state value for the first time. The settling time $t_{s}$ is the time when the step response enters and stays within the error range. The overshoot $\sigma_{p}$ is the ratio of the instantaneous maximum deviation value of the step response to the steady-state value. In addition, error integration metrics IAE, ISE, ITSE, *ITAE* are widely used. Among four error integration metrics, *ITAE* is measured by multiplying the time by the absolute value of the error integral [29], which is seen as one of the best performance metrics in single-parameter optimal control and adaptive control [30].

ITAE is selected as the objective function and a set of optimal (minimum *ITAE* value) PID parameters are tuned by metaheuristic algorithms. The *ITAE* calculation equation is given as Equation (6):

(6) $$ITAE=\int_{0}^{Time\_s}t\left|e\left(t\right)\right|dt$$

where Time_s is the time for the PID controller to reach stability.

### 2.2. CFHBA-PID Algorithm

#### 2.2.1. Honey Badger Algorithm (HBA)

The Honey Badger Algorithm (HBA) is inspired by the intelligent hunting behavior of honey badgers. HBA achieves the balance between exploration and exploitation [31] by setting appropriate randomness. In the HBA algorithm, the smell intensity of the prey I is related to the concentration strength of the prey S and the distance between the prey and the honey badger $d_{i}$, which enables search agents to transfer from exploration to exploitation and avoids falling into local optimum. The density factor α is a randomization control factor that decreases with time to reduce population diversity throughout iterations. This achieves the required trade-off balance between exploration and exploitation. F can provide population diversity in the search process by changing the search direction. HBA has two modes of locating food sources, which are digging mode and honey mode:

1. Digging mode: Honey badgers use their rat sniffing skills to slowly approach the prey and dig the prey in a cardioid way. In digging mode, the HBA position update process ($x_{new}$) is given as Equation (7):
  (7) $$x_{new}=x_{prey}+F\times \beta \times I\times x_{prey}\;+F\times r_{3}\times \alpha \times d_{i}\times \left|\cos\left(2\pi r_{4}\right)\times \left[1-\cos\left(2\pi r_{5}\right)\right]\right|$$
  where $x_{prey}$ is the global best position of the prey so far. I is the smell intensity of the prey. α is the update density factor. β is the ability of the honey badger to get food (default = 6). $r_{3}$, $r_{4}$, and $r_{5}$ are three different random numbers between 0 and 1. F is a flag to change the search direction, which is given as Equation (8):
  (8) $$F=\{\begin{array}{c} 1\;\;\;\;r_{6}\leq 0.5\\ -1\;\;\;r_{6}>0.5 \end{array}$$
  I is the smell intensity of the prey that is related to the concentration strength of the prey S and the distance between the prey and the honey badger $d_{i}$. The smell intensity of the prey I is strong when the concentration strength of the prey is high and the distance between the prey and the honey badger is close. Honey Badgers will search faster when the smell intensity of the prey is strong. I, S, and $d_{i}$ are given respectively using Equations (9)–(11):
  (9) $$I_{i}=r_{2}\times \frac{S}{4\pi d_{i}^{2}}$$
  (10) $$S={\left(x_{i}-x_{i+1}\right)}^{2}$$
  (11) $$d_{i}=x_{prey}-x_{i}$$
  where $r_{2}$ is a random number between 0 and 1. The density update factor α is a random variable that controls time-varying randomness, which can be used to achieve smooth transitions during exploration and development. It is given as Equation (12):
  (12) $$\alpha =C\times \exp\left(\frac{-t}{t_{max}}\right)$$
  where C is greater than or equal to 1 (default = 2). t is the current number of iterations and $t_{max}$ is the maximum number of iterations.
2. Honey mode: The honey badger likes eating bee larvae and pupae, and they cooperate with a honey-guide (a bird) to find bee larvae. It is difficult for the honey badger to find the hive, but its claws can easily break open the hive, while the honey-guide can quickly find the hive but cannot break it. Therefore, the honey-guide guides the honey badger toward the hive and finally shares the bee larvae. In honey mode, the HBA position update process ($x_{new}$) is given as Equation (7):
  (13) $$x_{new}=x_{prey}+F\times r_{7}\times \alpha \times d_{i}$$
  where $r_{7}$ is a random number between 0 and 1.

#### 2.2.2. Complementary Factor (CF)

For a complex PID control system, simply using *ITAE* as the fitness of the metaheuristic algorithms may make the rise time shorter, but the overshoot is not well controlled, which is reflected in Equation (6); furthermore, a large overshoot is fatal to the attitude control of the balancing robot, which causes serious impact and interference. Therefore, the complementary factor (*CF*) is proposed to achieve the fusion of *ITAE* and overshoot quantity, which leads to a new indicator, CF-ITAE. The CF-ITAE calculation equation and overshoot calculation equation are given as Equations (14) and (15):

(14) $$\mathrm{CF-ITAE}=CF\times ITAE+\left(1-CF\right)\times \sigma_{p}$$

(15) $$\sigma_{p}=\frac{c{\left(t\right)}_{max}-c\left(\infty \right)}{c\left(\infty \right)}\times 100\%\;$$

where CF is a constant between 0 and 1. Equation (15) shows that when c(∞) is a constant, the relationship between the overshoot $\sigma_{p}$ and the maximum value of the response $c{\left(t\right)}_{max}$ can be given as Equation (16):

(16) $$\sigma_{p}\propto c{\left(t\right)}_{max}$$

Therefore, we replace the overshoot $\sigma_{p}$ with $c{\left(t\right)}_{max}$. The improved CF-ITAE calculation equation is given as Equation (17):

(17) $$\mathrm{CF-ITAE}=CF\times ITAE+\left(1-CF\right)\times c{\left(t\right)}_{max}$$

CF determines the direction of CF-ITAE rectification. A larger CF indicates that the CF-ITAE has less confidence in the overshoot amount, while a smaller *CF* indicates that the CF-ITAE has more confidence in the overshoot amount. The complementary factor CF is implemented as a hyperparameter in this algorithm so that the metrics can be changed according to the actual requirements, and it achieves a balance between overshoot and rise time while retaining the original performance of *ITAE*.

Using CF-ITAE as the fitness, the value of the *CF* has an important influence on the search of the metaheuristic algorithm. If the weight of the algorithm for the overshoot is too small, and the algorithm may obtain a PID system with too much overshoot and very short rise time, we can change the search direction of the algorithm by reducing the value of the *CF* to obtain the desired solution. On the contrary, if the overshoot is small but the rise time is too long, the value of *CF* should be increased to increase the weight of the *ITAE* in the CF-ITAE in order to achieve the purpose of shortening the rise time of the tuning result.

Therefore, due to the simplicity and understandability of the CF-ITAE calculation in Equation (17), the user can easily understand the impact of *CF* and adjust it according to their requirements. If the user has high requirements for overshoot, the *CF* value can be reduced until the desired solution is obtained. It can be seen that compared with the ITAE, CF-ITAE can not only solve the problem of large overshoot, but also can be adjusted according to user needs.

#### 2.2.3. CFHBA-PID Algorithm

The CFHBA-PID algorithm combined the HBA algorithm with the Dual-loop PID algorithm by setting $K_{p\_inside}$, $K_{i\_inside}$, $K_{d\_inside}$, $K_{p\_outside}$, $K_{i\_outside}$, and $K_{d\_outside}$ of the Dual-loop PID algorithm as the search space of HBA algorithm. Two sets of PID parameters are obtained for each search, and the parameters obtained from the search are imported into the balanced robot model implemented by Simulink to calculate the *ITAE* value and the overshoot amount. Then the CF-ITAE can be calculated by Equation (17). The CF-ITAE metric is used as the fitness of the metaheuristic algorithm HBA to constrain the search direction to obtain the optimal solution.

**Figure 6:**
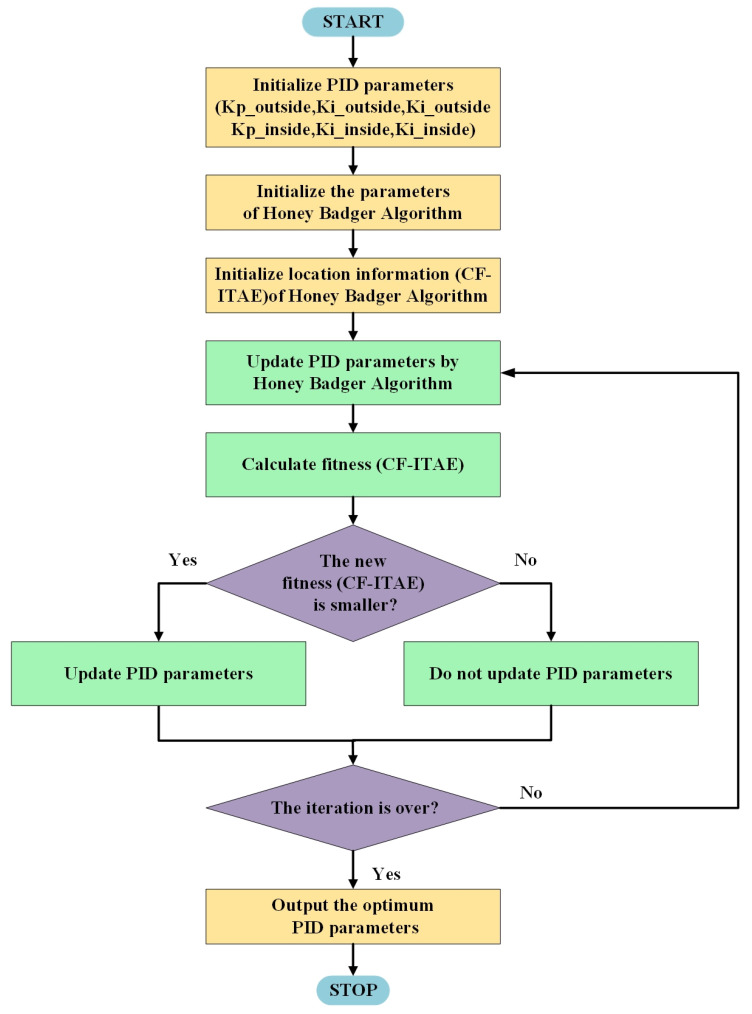
CFHBA-PID algorithm flow chart.

The CFHBA-PID control algorithm steps described by Figure 6 are:

Step 1: Initialize PID parameters. The six parameters $K_{p\_inside}$, $K_{i\_inside}$, $K_{d\_inside}$, $K_{p\_outside}$, $K_{i\_outside}$, and $K_{d\_outside}$ are used as the solution space, and the relevant parameters of HBA are initialized.

Step 2: The Dual-loop PID control algorithm calculates the fitness value (CF-ITAE) as the initial fitness, and the best fitness value and the corresponding PID parameters are recorded for subsequent updates.

Step 3: Start iterations and update the PID parameters by HBA for each iteration, and the new fitness (CF-ITAE) is calculated by the Dual-loop PID control algorithm.

Step 4: The calculated fitness value is compared with the previously recorded best fitness, and if the new fitness obtained is smaller, the fitness and its corresponding PID parameters will be updated.

Step 5: When the iteration is complete, the best fitness is output along with its corresponding PID parameters.

## 3. Experiments and Results

**Figure 7:**
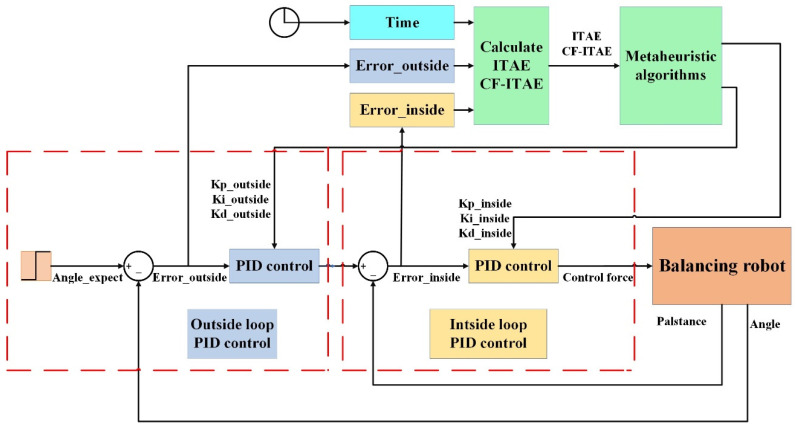
The Simulink model of Dual-loop PID attitude control of the balancing robot.

To verify the performance of the proposed algorithm, a series of experiments and comparisons are conducted, including comparing the HBA-PID algorithm with existing mainstream algorithms, and verifying the importance of complementary factors as well as testing the superiority of the CFHBA-PID algorithm. The Dual-loop PID attitude control of the balancing robot is used as the experimental object. The simulation experiments of the balancing robot were implemented by Simulink, and the Simulink model of the Dual-loop PID control algorithm for the attitude control of the balancing robot is shown in Figure 7. The relevant parameter values and ranges for the balancing robot are shown in Table 1, and the parameters related to the metaheuristic algorithm are set in Table 2.

**Figure 8:**
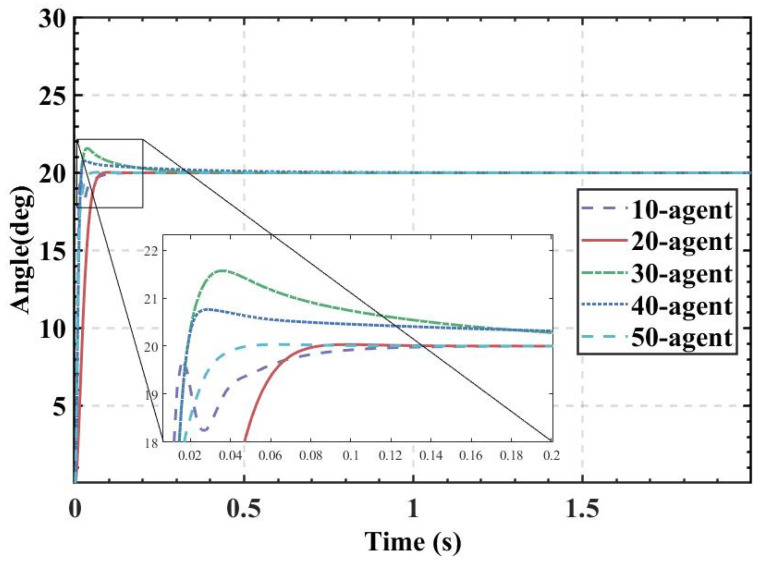
The actual angle of the balancing robot under HBA-PID algorithm.

**Figure 9:**
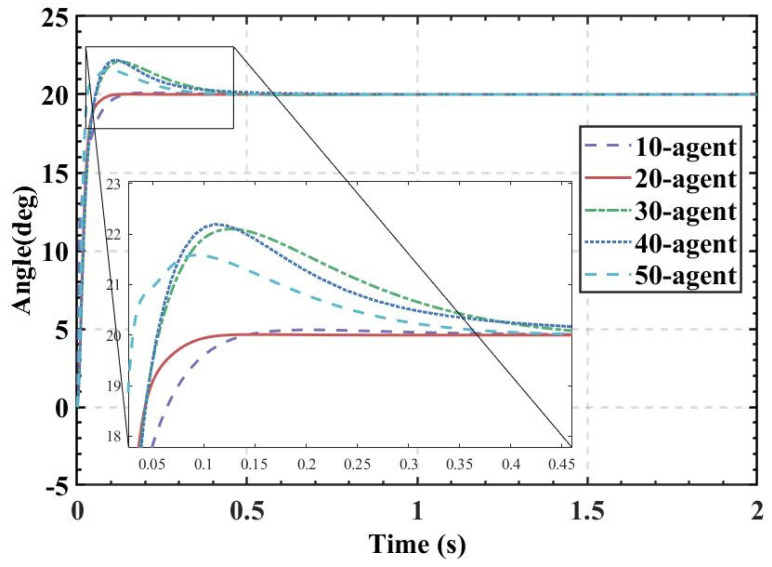
The actual angle of the balancing robot under AOA-PID algorithm.

**Figure 10:**
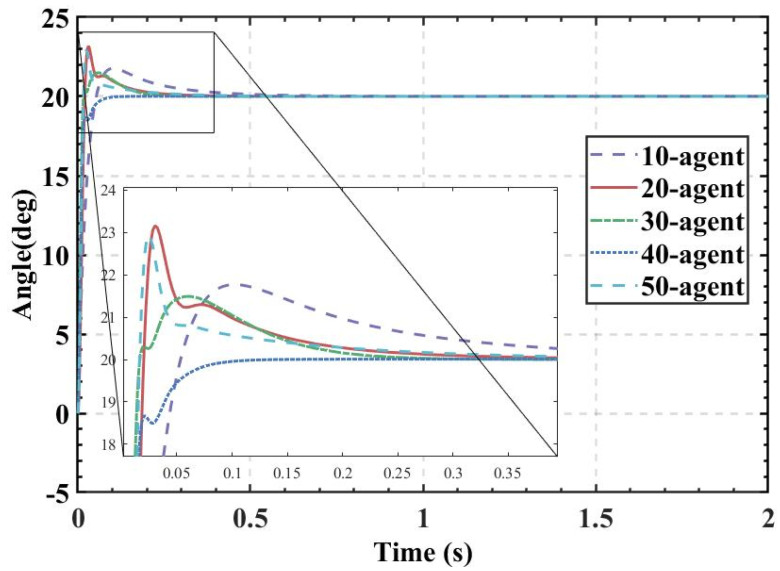
The actual angle of the balancing robot under WOA-PID algorithm.

**Figure 11:**
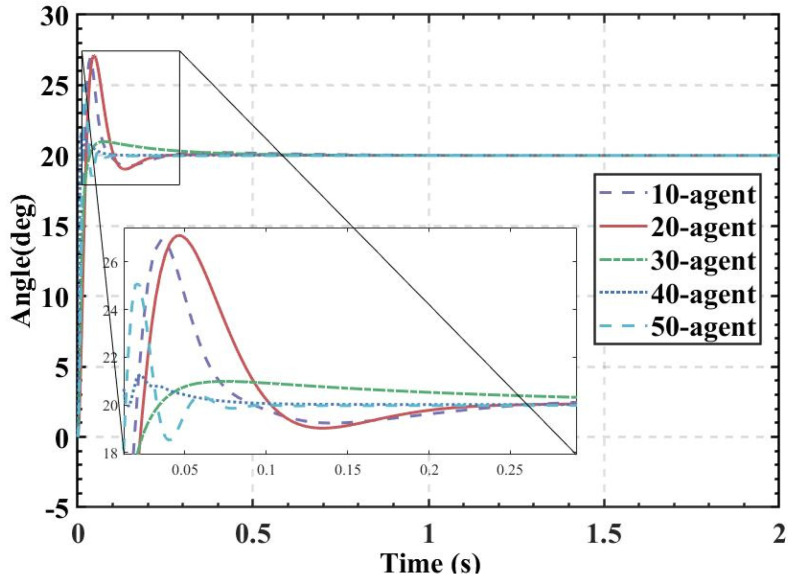
The actual angle of the balancing robot under PSO-PID algorithm.

**Figure 12:**
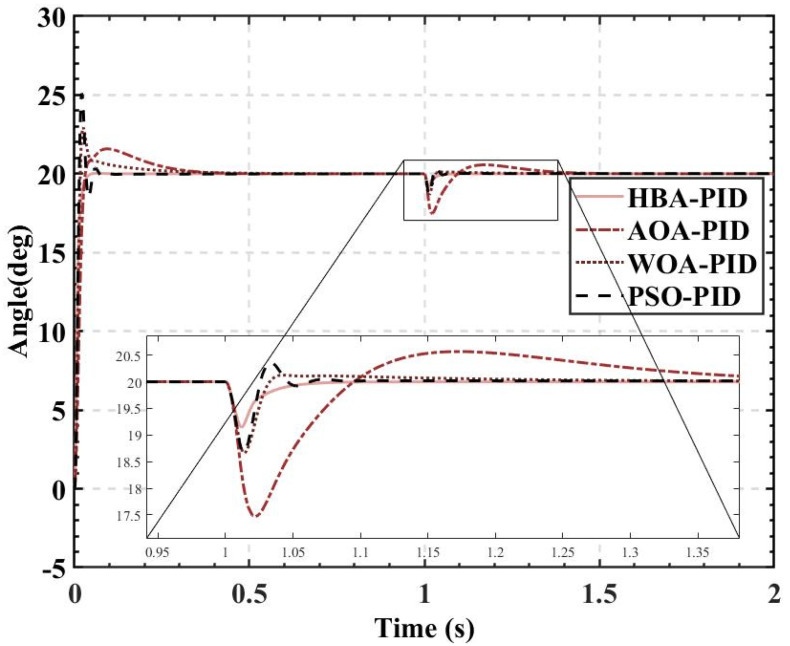
The actual angle of the balancing robot under HBA-PID, AOA-PID, WOA-PID, and PSO-PID algorithms after applying the external force.

**Figure 13:**
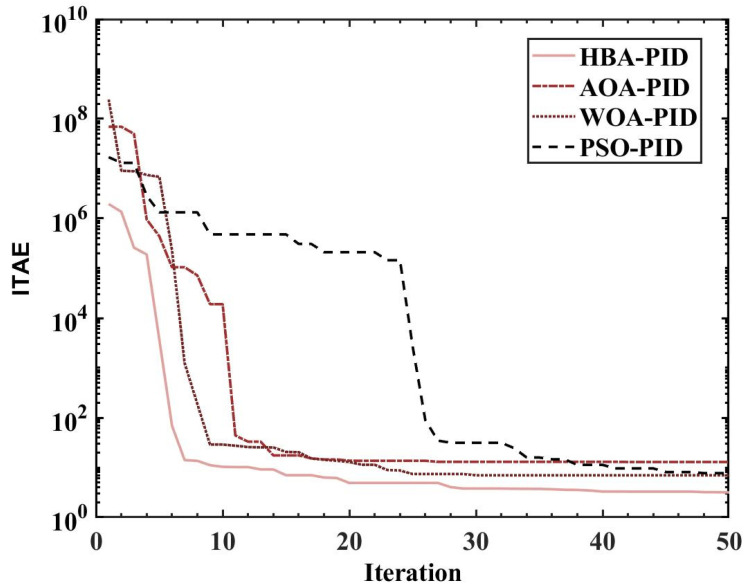
Fitness curves when optimal parameters are obtained by four metaheuristic algorithms.

ITAE is used as the fitness, and the population size is set to 10, 20, 30, 40, and 50. The fitness values, PID parameters, and time-domain metrics of the attitude control of the balancing robot obtained by the HBA-PID, AOA-PID, WOA-PID, and PSO-PID algorithms are shown in Table 3, Table 4, Table 5 and Table 6. The step responses of the actual angle of the balancing robot are shown in Figure 8, Figure 9, Figure 10 and Figure 11. In addition, to test the robustness of the algorithm when subjected to external disturbance, we apply an external force to break the balance of the balancing robot at a population size of 50, when time t = 1, and compare the robustness of the algorithm by observing the recovery speed and the magnitude of overshoot of the balancing robot. The actual angle of the balancing robot under HBA-PID, AOA-PID, WOA-PID, and PSO-PID algorithms after applying the external force is shown in Figure 12. The fitness value curves are shown in Figure 13.

**Figure 14:**
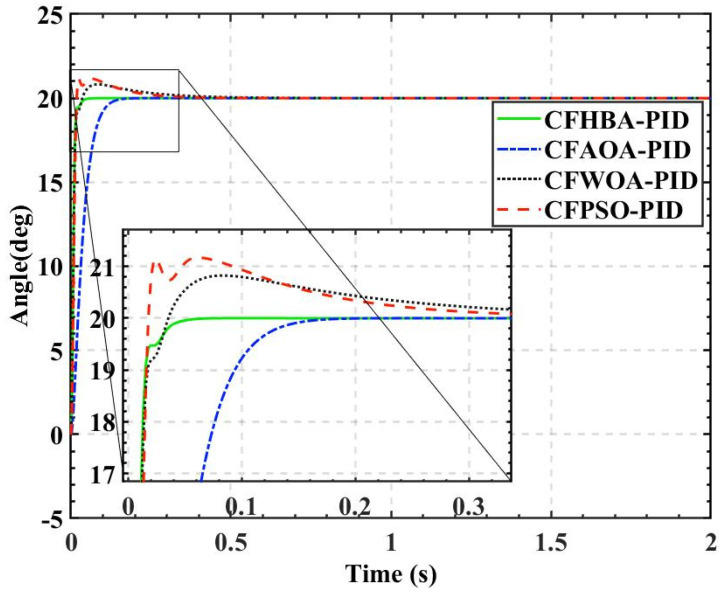
The actual angle of the balancing robot under metaheuristic algorithms and complementary factor.

**Figure 15:**
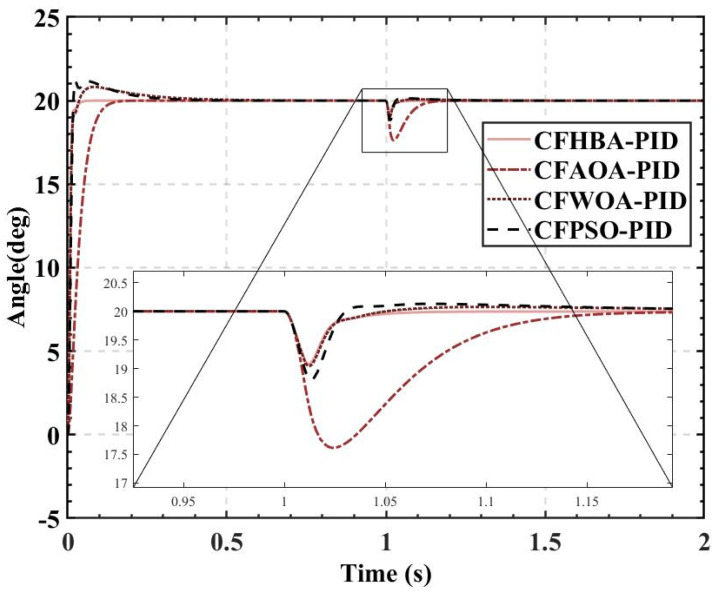
The actual angle of the balancing robot under CFHBA-PID, CFAOA-PID, CFWOA-PID, and CFPSO-PID algorithms after applying the external force.

**Figure 16:**
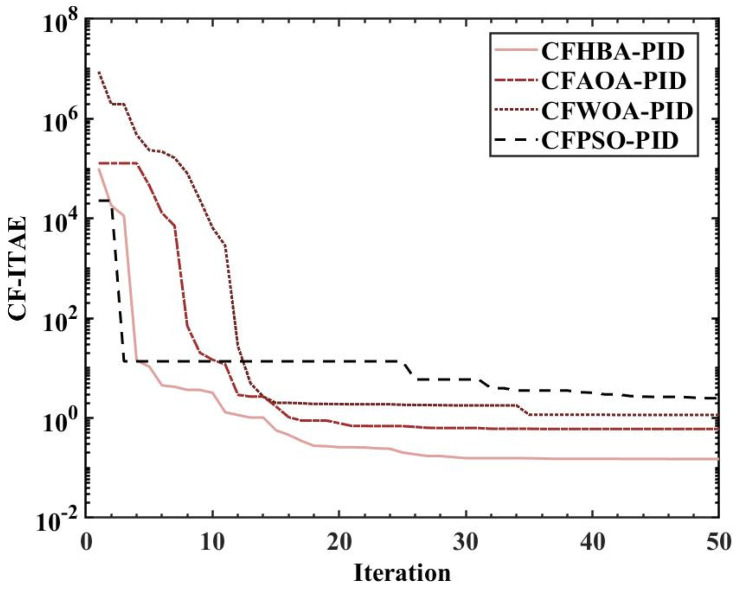
Fitness curves under different metaheuristic algorithms and complementary factor.

The overshoots obtained by the HBA-PID algorithm, AOA-PID algorithm, WOA-PID algorithm, and PSO-PID algorithm with a population size of 50 are 0.168%, 7.9185%, 14.6905%, and 25.3665%, respectively, while the rise times are kept short. It can be seen that using *ITAE* as the fitness, the rise time is preferred between the balance of overshoot and rise time. Since the amount of overshoot has a large impact on the attitude control of the balancing robot, the CF-ITAE indicator is proposed to achieve the fusion between *ITAE* and overshoot by using the CF. CF-ITAE enables the metaheuristic algorithm to obtain parameters that balance overshoot and rise time in the search for the optimal parameters. To verify the effectiveness of CF-ITAE, we compare the PID parameters and the time-domain metrics of the attitude control of the balancing robot obtained by combining HBA-PID, AOA-PID, WOA-PID, and PSO-PID algorithms with CF-ITAE. The *CF* is 0.05, the number of populations is 50, and other parameters are set as before. The data are shown in Table 7, and the step response of the actual angle of the balancing robot is shown in Figure 14. In order to test the robustness of the algorithm when subjected to external disturbance, we apply an external force to break the balance of the balancing robot at a population size of 50, when time t = 1, and compare the robustness of the algorithm by observing the recovery speed and the magnitude of overshoot of the balancing robot. The actual angle of the balancing robot under CFHBA-PID, CFAOA-PID, CFWOA-PID, and CFPSO-PID algorithms after applying the external force is shown in Figure 15, and the corresponding fitness curve is shown in Figure 16.

**Figure 17:**
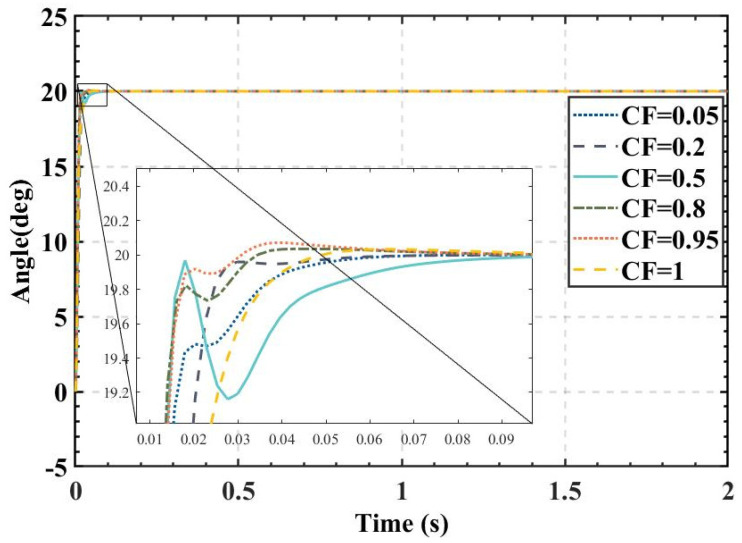
Actual angle of the balancing robot under CFHBA-PID algorithm with different complementary factors.

In addition, to investigate the effect of the value of the *CF* on the performance of the CFHBA-PID algorithm, *CF* is set to 0.05, 0.20, 0.50, 0.80, 0.95, and 1 for the experiments to obtain the PID parameters as well as the time-domain indicators of the attitude control of the balancing robot. The obtained data are shown in Table 8, and the step responses of the actual angle of the balancing robot are shown in Figure 17.

## 4. Discussion

As is indicated in Table 3, Table 4, Table 5 and Table 6 and Figure 8, Figure 9, Figure 10 and Figure 11, the most effective algorithms in balancing robot attitude control are expected to be a combination of the HBA-PID algorithm with a population size of 50, the AOA-PID algorithm with a population size of 20, the WOA-PID control algorithm with a population size of 40, and the PSO-PID control algorithm with a population size of 40. By comparing the optimal results obtained by the four algorithms (the bolded data in each table), we can easily find that the data obtained by the HBA-PID algorithm with a population size of 50 have the smallest stability time and *ITAE* value in balancing robot attitude control. From the above data, it can be seen that the *ITAE* values obtained by the HBA-PID algorithm are all less than 10, which means the results obtained by the HBA-PID are closer to the global optimal solution. Figure 12 shows that the HBA-PID algorithm produces the smallest angular change when disturbed by external force and also has the shortest stabilization time to recover to an error value of 2%, which shows that the HBA-PID algorithm has the best robustness for balancing robot attitude control among the four algorithms. The fitness curves obtained by the four metaheuristic algorithms are presented in Figure 13, indicating that the HBA-PID algorithm converges fastest in the search for the optimal PID parameters and obtains the smallest adaptation value, which also indicates that the HBA-PID algorithm outperforms other metaheuristic algorithms in balancing robot attitude control. Therefore, the proposed HBA-PID algorithm is better than other control algorithms in balancing robot attitude control.

Comparing the results of Table 7 and Figure 14 with the time-domain metrics obtained with *ITAE* as the fitness, it is found that the overshoot is effectively reduced by introducing the CF. The CFHBA-PID algorithm, CFAOA-PID algorithm, CFWOA-PID algorithm, and CFPSO-PID algorithm had 0.0035%, 0.0015%, 4.1095%, and 5.8615% overshoots, respectively, which were 0.1645%, 7.917%, 10.581%, and 19.505% less compared to the original algorithm, respectively. Figure 15 shows that CFHBA-PID algorithm, CFWOA-PID algorithm, and CFPSO-PID algorithm have good robustness and stability after introducing the CF. Considering the attitude control of the balancing robot by the three algorithms at 0–0.5 s, it is seen that the CFHBA-PID algorithm is the optimal algorithm in terms of robustness and stability.

Figure 16 shows that with the introduction of complementary factors, although the CFHBA-PID algorithm converges slightly less quickly than the CFPSO-PID algorithm in the search for the optimal parameters, the CFHBA-PID algorithm obtains the smallest fit- ness value and outperforms the other three algorithms overall. Therefore, the proposed CFHBA-PID algorithm could be considered as the most effective in balancing the robot’s attitude control and is superior to other algorithms.

The new rectification indicator CF-ITAE is obtained by using *CF* for the fusion of *ITAE* and overshoot. *CF* is used as a hyperparameter to play a role in the parameter tuning of the system to balance the relationship between the overshoot amount and the rise time, and the *CF* value can be adjusted according to the demand of the system, so that the system can achieve better results.

Table 8 and Figure 17 show that when the CFs are 0.80 or 0.95, the CFHBA-PID algorithm does not have enough weight on the overshoot amount and does not achieve a significant effect, and when the *CF* is 0.5, the CFHBA-PID algorithm increases the weight on the overshoot amount, so that the algorithm can effectively reduce the overshoot amount in the attitude control of the balancing robot. The overshoots are less than 0.01% for the *CF*s of 0.05, 0.2, and 0.5, indicating that the CFHBA-PID algorithm has effectively controlled the overshoot. It can be seen that when the *CF* is less than or equal to 0.5 as a hyperparameter, the overshoot in balancing robot attitude control is negligible, and the effect is significant for balancing robot attitude control at this time. Therefore, when the CFHBA-PID control algorithm is applied in the attitude control of the balancing robot, the best effect is achieved when the *CF* is less than 0.5.

## 5. Conclusions

In the present paper, the CFHBA-PID algorithm is proposed and implemented to the balancing robot attitude control. The HBA-PID algorithm is compared with the AOA-PID algorithm, WOA-PID algorithm, and PSO-PID algorithm through experimental simulation. The experimental results show that the HBA-PID algorithm outperforms the other three algorithms in terms of overshoot, stabilization time, *ITAE* value, and convergence speed of fitness value, which proves that the HBA-PID algorithm has the best control effect in the attitude control of the balancing robot. In addition, the complementary factor (CF) is introduced to the algorithm, and CF-ITAE is proposed to perform the fusion between the overshoot and *ITAE* to achieve the balance between the overshoot and rise time. Compared with the CFHBA-PID algorithm, CFAOA-PID algorithm, CFWOA-PID algorithm, and CFPSO-PID algorithm, the overshoot of the CFHBA-PID algorithm is very close to that of the CFAOA-PID algorithm; meanwhile, the CFHBA-PID algorithm is better than the other three algorithms in terms of rise time, stability time, and CF-ITAE value.

HBA is applied to PID parameter tuning in balancing robot attitude Dual-loop control for the first time, and the complementary factors are introduced to propose the CFHBA-PID algorithm, which is the most effective in balancing robot attitude control when compared with other existing algorithms. Therefore, the CFHBA-PID algorithm is suggested to be applied into other complex control systems in future work.

## Figures and Tables

**Table 1:** Parameters related to balancing robot attitude control.

Elements	Value
$K_{p\_inside}$ $,\;K_{i\_inside}$ $,\;K_{d\_inside}$ $,\;K_{p\_outside}$ $,\;K_{i\_outside}$ $,\;K_{d\_outside}$	(0,20]
Simulation time	2 s
Initial angle	0 deg
Expected angle (balanced angle)	20 deg
Iterations	50
Coefficient of static friction	0.5
Coefficient of dynamic friction	0.3
Damping	1 × 10^3^ N/(m/s)

**Table 2:** Parameters related to metaheuristic algorithm.

Algorithms	Parameters
HBA	β=6 , C=2
AOA	$C_{1}=2$ $,\;C_{2}=6$ $C_{3}=1,\;C_{4}=2$
WOA	a variable decreases linearly from 2 to 0 (Default) a_2_ linearly decreases from −1 to −2 (Default)
PSO	Inertia weight decreases linearly from 0.9 to 0.4 (Default) C_1_ (individual-best acceleration factor) increases linearly from 0.5 to 2.5 (Default) C_2_ (global-best acceleration factor) decreases linearly from 2.5 to 0.5 (Default)

**Table 3:** Attitude control of balancing robots under HBA-PID algorithm.

Agents	Parameters $(K_{p\_outside}$$,\;K_{i\_outside}$$,\;K_{d\_outside}$, $K_{p\_inside}$$,\;K_{i\_inside}$$,\;K_{d\_inside}$)	Overshoot of Angle	Rise Time of Angle	Settling Time of Angle	ITAE
10	(1.3567, 2.7261 × 10^−2^, 1.1688 × 10^−2^, 18.8397, 6.5175 × 10^−1^, 2.2346 × 10^−2^)	0.0030%	0.0119 s	0.0589 s	7.1655
20	(6.9749 × 10^−1^, 9.9871 × 10^−4^, 1.2302 × 10^−3^, 8.3548, 3.7914, 5.1196 × 10^−3^)	0.1570%	0.0477 s	0.0633 s	6.3359
30	(1.6662, 14.4183, 4.2575 × 10^−4^, 20, 1.3909 × 10^−2^, 5.4942 × 10^−2^)	7.8405%	0.0146 s	0.1587 s	6.3168
40	(1.7247, 6.0925, 3.7071 × 10^−4^, 20, 3.0678 × 10^−2^, 2.8151 × 10^−2^)	3.0890%	0.0146 s	0.1358 s	6.6401
50 *	**(1.5021, 9.7897 × 10^−3^, 3.5043 × 10^−3^,** 20, 1.7089 × 10^−2^, 9.1596 × 10^−2^) *	**0.1680%**	**0.0160 s**	**0.0295 s**	**3.1658**

* Where bold indicates the best parameters.

**Table 4:** Attitude control of balancing robots under AOA-PID algorithm.

Agents	Parameters $(K_{p\_outside}$$,\;K_{i\_outside}$$,\;K_{d\_outside}$, $K_{p\_inside}$$,\;K_{i\_inside}$$,\;K_{d\_inside}$)	Overshoot of Angle	Rise Time of Angle	Settling Time of Angle	ITAE
10	(3.4948 × 10^−1^, 8.5349 × 10^−3^, 1.3432 × 10^−2^, 13.2434, 1.4101 × 10^−2^, 3.9182 × 10^−1^)	5.2200%	0.0533 s	0.1003 s	13.5278
20 *	**(7.4634 × 10^−1^** **, 1.8467 × 10^−2^** **, 1.6600 × 10^−2^,** **3.2854, 4.3815 × 10^−2^, 1.8112 × 10^−2^** **)**	**0.0645%**	**0.0379 s**	**0.0683 s**	**12.9221**
30	(7.9081 × 10^−1^, 3.0156, 2.7857 × 10^−2^, 1.5723, 9.7823 × 10^−3^, 1.6325 × 10^−2^)	10.4960%	0.0399 s	0.3553 s	21.0364
40	(8.1478 × 10^−1^, 2.8729, 1.7204 × 10^−2^, 2.0965, 5.1542 × 10^−2^, 2.5170 × 10^−2^)	10.9575%	0.0405 s	0.3440 s	17.7394
50	(9.1902 × 10^−1^, 6.0744, 1.8935 × 10^−2^, 3.9686, 1.5964 × 10^−2^, 2.6454 × 10^−2^)	7.9185%	0.0241 s	0.2620 s	13.1745

* Where bold indicates the best parameters.

**Table 5:** Attitude control of balancing robots under WOA-PID algorithm.

Agents	Parameters $(K_{p\_outside}$$,\;K_{i\_outside}$$,\;K_{d\_outside}$, $K_{p\_inside}$$,\;K_{i\_inside}$$,\;K_{d\_inside}$)	Overshoot of Angle	Rise Time of Angle	Settling Time of Angle	ITAE
10	(6.8813 × 10^−1^, 3.6046, 3.6926 × 10^−4^, 20, 15.0197, 4.5869 × 10^−1^)	8.8530%	0.0399 s	0.3264 s	72.5270
20	(1.3457, 6.5516, 3.3192 × 10^−2^, 2.9791, 2.7506 × 10^−1^, 1.9495 × 10^−2^)	15.8170%	0.0176 s	0.1673 s	17.7998
30	(1.4324, 20, 7.2739 × 10^−3^, 20, 7.6224 × 10^−1^, 2.5866 × 10^−2^)	7.4650%	0.0129 s	0.1549 s	7.6531
40 *	**(1.2947, 1.2293 × 10^−1^** **, 6.5177 × 10^−3^,** **20, 5.1214, 2.5887 × 10^−3^** **)**	**0.0995%**	**0.0163 s**	**0.0578 s**	**6.9713**
50	(1.8339, 11.7589, 3.1272 × 10^−3^, 12.2438, 1.6145 × 10^−1^, 3.1801 × 10^−2^)	14.6905%	0.0123 s	0.1495 s	8.5643

* Where bold indicates the best parameters.

**Table 6:** Attitude control of balancing robots under PSO-PID algorithm.

Agents	Parameters $(K_{p\_outside}$$,\;K_{i\_outside}$$,\;K_{d\_outside}$, $K_{p\_inside}$$,\;K_{i\_inside}$$,\;K_{d\_inside}$)	Overshoot of Angle	Rise Time of Angle	Settling Time of Angle	ITAE
10	(2.0667, 8.1296, 6.5740 × 10^−2^, 1.4035, 14.8514, 7.3100 × 10^−3^)	34.7265%	0.0183 s	0.1974 s	61.5569
20	(2.1407, 20, 4.5500 × 10^−3^, 1.9109, 9.8152, 3.9350 × 10^−2^)	35.5830%	0.0230 s	0.1687 s	50.6170
30	(1.1807, 5.8597, 4.0200 × 10^−3^, 14.8726, 2.9790 × 10^−2^, 6.2500 × 10^−2^)	4.9460%	0.0212 s	0.2490 s	7.9056
40 *	**(2.7637, 1.1961, 2.2400 × 10^−3^,** **20, 1.0920 × 10^−2^, 2.4012 × 10^−1^** **)**	**8.2030%**	**0.0066 s**	**0.0515 s**	**7.7164**
50	(2.5067, 1.9125 × 10^−1^, 1.2700 × 10^−3^, 13.6994, 20, 3.0000 × 10^−4^)	25.3665%	0.0122 s	0.0507 s	13.3757

* Where bold indicates the best parameters.

**Table 7:** Attitude control of balancing robots under metaheuristic algorithms and complementary factor.

**Algorithms**	Parameters $(K_{p\_outside}$$,\;K_{i\_outside}$$,\;K_{d\_outside}$, $K_{p\_inside}$$,\;K_{i\_inside}$$,\;K_{d\_inside}$)	**Overshoot of Angle**	**Rise Time of Angle**	**Settling Time of Angle**	**ITAE**
CFHBA-PID	(1.6718, 1.0090 × 10^−2^, 3.9545 × 10^−3^, 19.9632, 4.3772 × 10^−4^, 5.0410 × 10^−2^)	0.0035%	0.0131 s	0.0288 s	0.1511
CFAOA-PID	(4.8467 × 10^−1^, 2.1000 × 10^−3^, 6.1906 × 10^−4^, 8.0550, 6.5689 × 10^−1^, 2.4953 × 10^−2^)	0.0015%	0.0757 s	0.1157 s	0.6036
CFWOA-PID	(1.3836, 8.3422, 6.7865 × 10^−3^, 20, 2.4392, 3.688 × 10^−2^)	4. 1095%	0.0139 s	0.1854 s	1.1521
CFPSO-PID	(1.4725, 12.9553, 4.7766 × 10^−3^, 17.0569, 13.7370, 4.9587 × 10^−3^)	5.8615%	0.0148 s	0.1847 s	2.4918

**Table 8:** Attitude control of balancing robots under CFHBA-PID algorithm with different complementary factors.

**CF**	Parameters $(K_{p\_outside}$$,\;K_{i\_outside}$$,\;K_{d\_outside}$, $K_{p\_inside}$$,\;K_{i\_inside}$$,\;K_{d\_inside}$)	**Overshoot of Angle**	**Rise Time of Angle**	**Settling Time of Angle**
0.05	(1.6718, 1.0090 × 10^−2^, 3.9545 × 10^−3^, 19.9632, 4.3772 × 10^−4^, 5.0410 × 10^−2^)	0.0035%	0.0131 s	0.0288 s
0.20	(1.5286, 7.7354 × 10^−3^, 5.4841 × 10^−4^, 20, 7.0650 × 10^−2^, 4.1545 × 10^−3^)	0	0.0173 s	0.0226 s
0.50	(1.6431, 1.1681 × 10^−2^, 5.9419 × 10^−3^, 20, 2.9843 × 10^−2^, 2.0623 × 10^−2^)	0.0005%	0.0116 s	0.0383 s
0.80	(1.8229, 1.2032 × 10^−2^, 3.41784 × 10^−3^, 20, 9.1008 × 10^−5^, 6.3091 × 10^−2^)	0.1785%	0.0120 s	0.0159 s
0.95	(1.8367, 1.1547 × 10^−2^, 2.5180 × 10^−3^, 19.9999, 4.0450 × 10^−4^, 5.8050 × 10^−2^)	0.3610%	0.0125 s	0.0160 s
1	(1.5021, 9.7897 × 10^−3^, 3.5043 × 10^−3^, 20, 1.7089 × 10^−2^, 9.1596 × 10^−2^)	0.1680%	0.0160 s	0.0295 s
